# Obstructive Sleep Apnea and Parkinson’s Disease: Bidirectional Clinical and Pathophysiologic Links

**DOI:** 10.3390/ijms26083762

**Published:** 2025-04-16

**Authors:** Jia Dong James Wang, Nevin Yi Meng Chua, Ling-Ling Chan, Eng-King Tan

**Affiliations:** 1 Lee Kong Chian School of Medicine, Nanyang Technological University, Singapore 308232, Singapore; jwang082@e.ntu.edu.sg (J.D.J.W.); nchua013@e.ntu.edu.sg (N.Y.M.C.); 2 Departments of Neurology and Neuroradiology, Singapore General Hospital Campus, National Neuroscience Institute, Singapore 168581, Singapore; chan.ling.ling@singhealth.com.sg; 3 Neuroscience and Behavioural Disorders, Duke-NUS Medical School, Singapore 169857, Singapore

**Keywords:** Parkinson’s disease, obstructive sleep apnea, neurodegeneration, intermittent hypoxia, α-synuclein, sleep fragmentation

## Abstract

Obstructive sleep apnea (OSA) and Parkinson’s disease (PD) are highly prevalent conditions with significant global health impacts. OSA affects 17–34% of middle-aged adults, while more than 10 million worldwide have PD. Clinical studies demonstrate a bidirectional relationship, with the OSA prevalence being higher among PD patients suggesting that hypoxia and sleep fragmentation contribute to worsening motor and cognitive symptoms. Conversely, PD-associated neurodegeneration impairs respiratory control, exacerbating OSA. Diagnostic differentiation is particularly challenging due to overlapping symptoms, such as sleep disturbances, cognitive decline, and autonomic dysfunction. Emerging neuromodulation therapies, including deep brain stimulation and hypoglossal nerve stimulation, show dual therapeutic potential. The interplay between OSA and PD draws attention to the need for integrated diagnostic and therapeutic approaches. Additional longitudinal studies to evaluate their cause–effect relationship and identify neuroimaging and biochemical biomarkers to elucidate novel pathophysiologic clues can potentially identify novel therapeutic targets.

## 1. Introduction

Parkinson’s disease (PD) and obstructive sleep apnea (OSA) are highly prevalent chronic diseases that contribute significantly to the global health burden. OSA was reported to affect approximately 34% and 17% of middle-aged men and women worldwide [1]. PD affects more than 10 million people globally, with this number expected to double in the next 25 years [2,3]. As the global population ages, the incidence of both conditions is expected to rise, further amplifying their impact on public health.

The existence of etiologic links between OSA and PD has been debated. Observational studies [4] have shown that PD patients have a 1.92 times higher incidence of OSA, while patients with OSA have a 1.54 times higher incidence of PD. Here, we provide a concise summary of the clinical association and pathophysiological links between the two conditions and their therapeutic implications. In addition, we identify gaps in knowledge and limitations of current studies, and discuss potential strategies to address these challenges.

## 2. Pathophysiology of PD and OSA

Several mechanisms have been postulated to link OSA and PD. Intermittent hypoxia (IH) induced by OSA promotes oxidative stress, inflammation, and neuronal apoptosis [5]. This process involves reactive oxygen species (e.g., superoxide and hydrogen peroxide), which damage neurons and trigger the release of pro-inflammatory cytokines like TNF-α, IL-1β, and IL-6 [6]. These molecular alterations were thought to promote α-synuclein accumulation in the substantia nigra by influencing its oligomerization and fibril formation.

Furthermore, sleep fragmentation and disrupted Rapid Eye Movement (REM) sleep impaired the glymphatic system’s ability to clear α-synuclein [7]. This impairment is associated with alterations in aquaporin-4 water channel expression and the disruption of tight junction proteins, crucial for glymphatic function. The resulting accumulation of α-synuclein triggers astroglial and microglial inflammation, thus accelerating dopaminergic neuronal death. Additionally, reduced sympathovagal balance during REM sleep, a marker of autonomic dysfunction in OSA, was associated with a two-fold increase in PD risk [8]. This was underpinned by changes in muscarinic and nicotinic acetylcholine receptor expression in the brainstem as well as alterations in voltage-gated calcium channel function [9].

Conversely, PD can worsen OSA due to α-synuclein and neurofilament buildup in the brainstem (i.e., medulla and pontine respiratory centers) by disrupting autonomic control of breathing [10]. Dopaminergic degeneration diminishes ventilatory drive and impairs the brain’s response to hypoxia [11]. At the molecular level, this involves alterations in dopamine synthesis, release, and reuptake mechanisms. Cholinergic deficits, resulting from the vulnerability of cholinergic neurons to hypoxia, further destabilize REM sleep and disrupt normal respiratory patterns, potentially leading to a positive feedback mechanism driving OSA and PD progression.

Emerging research utilizing chronic intermittent hypoxia (IH) models has identified mitochondrial protein acetylation as a distinct pathway linking obstructive sleep apnea (OSA) to Parkinson’s disease (PD) progression [12]. While oxidative stress and α-synuclein aggregation are established hallmarks of neurodegeneration, IH uniquely induces compartment-specific acetylation through various mechanisms.

One such mechanism involves the enzymatic acetylation of COX17 at lysine residues K18 and K30 by the MOF–KANSL complex, disrupting complex IV assembly and impairing copper chaperoning [13]. Another mechanism entails pH-dependent, non-enzymatic acetylation of pyruvate dehydrogenase E1α (PDHE1α) at lysine 321, reducing pyruvate flux into the Krebs cycle; this modification is reversible via SIRT3-mediated deacetylation [14].

Addressing these modifications has shown varying degrees of reversibility: SIRT3 activation restores PDHE1α activity, while continuous positive airway pressure (CPAP) therapy enhances mitochondrial respiration but has limited efficacy on MOF-dependent COX17 acetylation.

Additionally, IH-induced endoplasmic reticulum (ER) stress activates the ATF4/CHOP pathway [15], amplifying neuroinflammation and apoptosis through the suppression of Bcl-2 and upregulation of caspase-3. Notably, models of PD-associated LRRK2 mutations [16,17] exhibit minimal overlap with IH-induced acetylation targets in electron transport chain subunits, highlighting OSA’s unique metabolic fingerprint.

**Figure 1:**
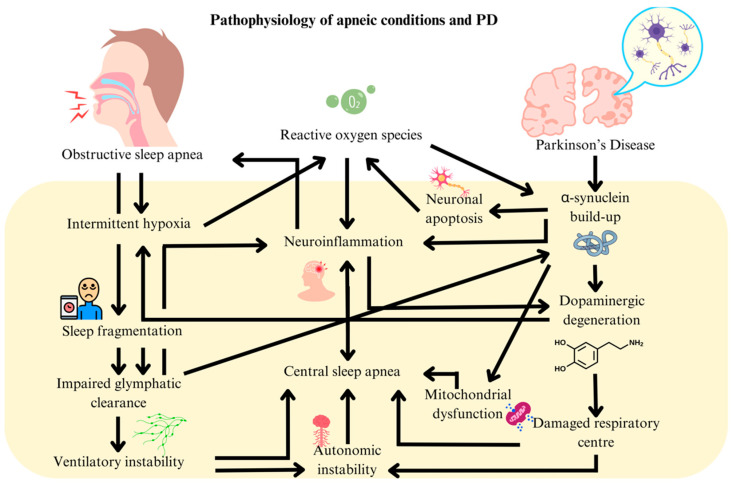
Pathophysiological links between obstructive sleep apnea and Parkinson’s disease.

These findings position mitochondrial acetylation as a critical mechanism in hypoxia-driven neurodegeneration, where enzymatic (MOF/COX17) and chemical (PDHE1α) modifications converge to disrupt energy metabolism. This self-sustaining cycle arises from OSA-induced mitochondrial acetylation, glymphatic failure, and PD-mediated brainstem pathology exacerbating hypoxia (Figure 1). Bidirectional reinforcement occurs through neuroinflammation, autonomic dysfunction, and shared oxidative stress, with OSA’s distinct metabolic targets highlighting unique intervention points. Collectively, these mechanisms fuel a cascade of neurodegeneration wherein hypoxia, proteinopathy, and respiratory instability perpetually amplify one another.

## 3. Clinical Evidence Linking OSA and PD

Clinical evidence reveals a robust association between OSA and PD, with studies reporting nearly double the prevalence of OSA among PD patients compared to the general population. For instance, an analysis of 29,469 OSA patients from the Korean National Health Information Database [4], along with a meta-analysis [18] encompassing 93,332 patients, revealed a significantly elevated hazard ratio (HR) for PD development in OSA patients (hazard ratio (HR) 1.92) and vice versa (HR 1.59).

OSA has been shown to significantly exacerbate motor symptoms in PD [19]. This is evidenced by 20% higher Unified Parkinson’s Disease Rating Scale (UPDRS) scores in PD patients with comorbid OSA. Importantly, higher AHI scores correlated with worsening motor dysfunction. This relationship highlights the role of intermittent hypoxia and oxygen desaturation in accelerating neurodegeneration. Conversely, PD motor manifestations, such as impaired pharyngeal muscle coordination and diminished neural respiratory control worsened OSA by promoting upper airway dysfunction and collapse during sleep. This bidirectional relationship is reflected in worsening AHI levels and polysomnographic data showing an average increase of two apnoeic episodes/h in PD patients.

Cognitive symptoms demonstrate a bidirectional interplay between OSA and PD [20]. It was observed in recent studies involving 783 patients that OSA exacerbated PD-related cognitive decline. Furthermore, researchers [21] found that PD patients with moderate to severe cognitive impairment had significantly higher AHI scores compared to those with mild cognitive impairment (OR 9.38, 95% CI 2.53–160). These findings suggest that cognitive dysfunction in PD intensifies OSA. A list of other relevant clinical studies are summarized in Table S1.

## 4. Therapeutic Options for the Intersection of OSA and PD

Limited research has investigated continuous positive airway pressure (CPAP) therapy on PD patients with OSA. While CPAP has been shown to improve sleep quality and reduce apnea severity [22], its effects on PD-related motor symptoms have been inconsistent. Furthermore, treatments targeting PD, like levodopa, were found to possibly increase the severity and prevalence of OSA [23,24]. These agents, by enhancing dopaminergic signaling, are hypothesized to impair pharyngeal muscle tone or disrupt sleep architecture, potentially increasing airway collapsibility.

Newer neuromodulation therapies have shown promise in targeting both conditions but are relatively understudied. Studies [25,26] using deep brain stimulation (DBS) and repetitive Transcranial Magnetic Stimulation (rTMS) have demonstrated improved motor symptoms in PD, and enhanced sleep quality by increasing slow-wave activity and reducing arousals. These improvements are reflected by lower AHI scores and reduced likelihood of upper airway instability. Recent evidence also highlights the potential of hypoglossal nerve stimulation (HNS) as a treatment for PD. Biogenic amine deficiency in PD disrupts hypoglossal nerve function, contributing to respiratory dysfunction. Retrospective analyses of OSA interventions like HNS often exclude PD-related pharyngeal collapse patterns, such as retrognathia, which are present in 28% of PD patients and may limit therapeutic generalizability [24]. While biophysical models suggest HNS could improve lingual rigidity in PD, these studies neglect α-synuclein’s disruption of mitochondrial pathways, including synaptic vesicle acidification critical to axonal conductivity. Preclinical evidence demonstrates that α-synuclein oligomers reduce mitochondrial complex I activity, potentially blunting HNS efficacy [27]. Current PD trials lack integration of hypoxia-sensitive biomarkers (e.g., phosphorylated α-synuclein) and dynamic assessments of hypoglossal–levator palatini coordination during dopaminergic medication cycles. These gaps persist despite evidence that intermittent hypoxia exacerbates neuroinflammation and oxidative stress [28]. Future studies should prioritize PD-specific phenotyping and mechanistic validation to advance HNS as a dual-target therapy.

Another intervention is oxygen therapy, which shows promise in addressing hypoxia-induced damage in OSA and PD. Chronic intermittent hypoxia increases reactive oxygen species, triggering neuroinflammation and exacerbating dopaminergic neurodegeneration. Oxygen supplementation could stabilize mitochondrial function, reduce oxidative stress, and protect cholinergic and dopaminergic neurons. It may also preserve blood–brain barrier integrity, preventing neurotoxic infiltration and systemic inflammation. While CPAP and neuromodulation remain primary treatments, oxygen therapy could serve as an adjunctive intervention to counteract hypoxia-driven neurodegeneration, potentially improving OSA and PD symptoms. Further research is needed to elucidate its effects on α-synuclein aggregation and other molecular pathways linking OSA and PD [29].

## 5. Diagnostic Overlap Between Sleep Apnea and PD

The association between OSA and PD necessitates careful differentiation of their overlapping symptoms, including fatigue, cognitive decline, sleep disturbances, bradykinesia, mood disorders, and autonomic dysfunction due to their distinct treatment approaches. This diagnostic challenge arises from difficulty in determining whether these symptoms are primarily driven by OSA or neurodegenerative processes of PD. For example, cognitive dysfunction is common in both conditions, with OSA-induced hypoxia potentially exacerbating PD-related cognitive decline. Similarly, early-stage PD often manifests with non-motor symptoms like excessive daytime sleepiness, which are hallmark features of untreated OSA.

Given these overlapping manifestations, there is an urgent need for improved biomarkers and refined diagnostic criteria to facilitate differentiation between sleep disturbances originating from OSA or PD. While polysomnography remains a valuable tool, it often falls short in clarifying the underlying etiology. Combining full neurocognitive assessments in different cognitive, executive, and social domains with sleep studies could yield deeper insights, and this can be incorporated into routine clinical protocols. Comprehensive evaluations encompassing sleep architecture and neurocognitive function are crucial for accurate differentiation. There is also a need to identify common genetic, biochemical, and neuroimaging biomarkers that can help identify subsets of patients who are at risk of both conditions and those who are likely to progress. In addition, identifying diagnostic neuroinflammation and oxidative stress markers may help to facilitate disease monitoring and unravel pathophysiologic clues.

Central sleep apnea (CSA) adds complexity to the clinical profile by mimicking or coexisting with OSA, especially in neurodegenerative disorders. Distinguishing these conditions is critical, as CSA may result from PD-related neurodegeneration. Although polysomnography remains the diagnostic gold standard, its reliability in PD patients is hindered by movement artifacts and fragmented sleep. Artificial intelligence-based tools trained on sleep study data offer a promising solution, improving diagnostic accuracy by differentiating CSA from OSA even amidst these challenges. Such advancements could enable more precise and tailored management strategies, ensuring PD patients receive appropriate interventions for their specific sleep disturbances. The diagnostic complexities directly inform therapeutic challenges, as discussed below.

## 6. Lack of Effective Therapy Targeting Both OSA and PD

Despite increasing awareness of the co-occurrence of OSA and PD, effective dual-targeted therapies remain limited. CPAP, the gold standard for OSA treatment, reduces apneic events and improves sleep quality but shows uncertain efficacy in alleviating PD symptoms, further hindered by low adherence rates. Alternative therapies, such as mandibular advancement devices and hypoglossal nerve stimulation, offer potential options, but their impact on PD-related deficits needs to be further examined.

Pharmacological treatments for PD, like dopaminergic and anticholinergic agents, may exacerbate OSA by impairing upper airway muscle tone or disrupting sleep architecture. Similarly, neuromodulation techniques like vagus nerve stimulation (VNS), while promising for PD symptom management, may increase airway obstruction. Approaches like HNS, rTMS, and DBS show potential benefits in mitigating OSA but are currently understudied. This therapeutic complexity highlights the need for personalized, multimodal strategies that address both OSA and PD symptoms.

Protein acetylation has emerged as a promising therapeutic target at the intersection of obstructive sleep apnea (OSA) and Parkinson’s disease (PD), due to its pivotal role in regulating mitochondrial function. In both conditions, chronic intermittent hypoxia (IH) alters the acetylation status of key mitochondrial proteins involved in oxidative phosphorylation, the tricarboxylic acid (TCA) cycle, and glycolysis—pathways essential for maintaining cellular energy homeostasis. These aberrant acetylation patterns disrupt mitochondrial efficiency, suggesting that therapeutic modulation of acetylation could help restore metabolic balance. Preclinical studies using histone deacetylase (HDAC) inhibitors, such as sodium butyrate, have demonstrated cognitive improvement in animal models exposed to chronic IH. These findings support the potential of acetylation-targeting compounds as novel interventions for cognitive impairment associated with both OSA and PD [30].

Further studies using midbrain organoids display key PD features and show exacerbated pathology when exposed to IH, mimicking OSA conditions. The organoids exhibit altered expression of genes involved in mitochondrial function, oxidative stress response, and protein homeostasis under hypoxic conditions [31]. Studies using antioxidants and mitochondrial-targeted compounds have shown promise in mitigating the effects of intermittent hypoxia on dopaminergic neurons. These findings highlight the potential of midbrain organoids as a tool for screening therapeutics that could address both OSA-related intermittent hypoxia and PD pathology.

Future research should prioritize integrated approaches combining neuromodulation, optimized pharmacotherapy, and tailored OSA management to achieve balanced neurological and sleep-related outcomes with the end goal of comprehensive care for these interrelated conditions. Furthermore, omics technologies, including genomics, proteomics, and metabolomics, offer a powerful approach to identifying shared molecular pathways underlying both OSA and PD. By integrating multi-omics data, researchers can uncover novel biomarkers and therapeutic targets that address the pathophysiological overlap between these conditions.

## 7. Level of Evidence and Research Limitations

The evidence linking OSA and PD predominantly stems from case–control association studies, limiting causal inferences. Despite robust epidemiological associations linking OSA and PD (HR 1.54–1.92), causality remains limited by studies predominantly having case–control associated or retrospective designs. To strengthen evidence, OSA onset should be tracked in PD prodromal cohorts and mendelian randomization studies.

The widespread reliance on non-polysomnographic (non-PSG) metrics in studies examining OSA in PD introduces significant validity concerns. In PD populations, the presence of frequent movement artifacts and disrupted sleep architecture complicates the accurate scoring of respiratory events. Furthermore, home sleep apnea testing often lacks the resolution needed to distinguish between obstructive and central apneas—a crucial limitation, given the high prevalence of mixed apnea syndromes in PD. This diagnostic ambiguity poses challenges in interpreting whether worsening disease severity reflects progression of PD, exacerbation of OSA, or undiagnosed CSA.

Consequently, outcome measures in interventional studies may not accurately reflect improvements across both conditions. The development of dual-targeted therapies must ensure that observed benefits are attributable to concurrent improvements in both OSA and PD, rather than isolated effects. Moreover, common comorbidities—such as cardiovascular disease, depression, and cognitive impairment—are often underreported or inadequately controlled for, further confounding interpretation.

To address these gaps, prospective cohort studies and randomized controlled trials employing gold-standard PSG and rigorous phenotyping are urgently needed. Longitudinal studies will also be essential to unravel the temporal dynamics between OSA and PD progression. Future research should prioritize these methodologies to elucidate the bidirectional influence between the two conditions and assess the impact of OSA treatment on both motor and non-motor PD symptoms. Such insights will be instrumental in advancing personalized and mechanism-driven therapeutic strategies.

## 8. Brain Localization in OSA and PD

The neurotopographical underpinnings of OSA in PD remain insufficiently understood. While OSA impacts regions like the brainstem and hypothalamus, PD-related neurodegeneration adds complexity to this relationship. Advanced neuroimaging techniques, such as functional MRI, are essential to pinpoint brain regions affected by OSA in PD, shedding light on how OSA influences motor and non-motor symptoms. This understanding is pivotal for neuromodulation strategies, as identifying areas where neurodegeneration and hypoxia overlap can guide interventions that address PD symptoms without exacerbating OSA. Integrating neuroimaging insights with clinical outcomes could pave the way for precision medicine approaches to manage these intertwined conditions effectively.

## 9. Conclusions

The intricate interplay between OSA and PD underscores a critical but underexplored area in clinical research. Evidence suggests that OSA exacerbates motor and non-motor symptoms of PD, contributing to neurodegeneration through mechanisms of hypoxia-induced oxidative stress, neuroinflammation, and disrupted glymphatic clearance. Conversely, PD-associated neurodegenerative changes impair respiratory control and exacerbate OSA, creating a vicious cycle that accelerates the progression of both conditions.

Despite this interplay, significant gaps remain. Current diagnostic tools and treatment modalities, including CPAP and pharmacological interventions, show promise but lack conclusive evidence of efficacy in addressing the intertwined pathophysiology. Emerging technologies, like neuromodulation, hold potential for diagnosis and treatment but require further validation. Future research should prioritize longitudinal studies, innovative biomarkers, and integrated therapeutic strategies to disentangle the complexities of this intersection and improve patient outcomes.

## Data Availability

No original data were collected in the course of this study.

## Supplementary Materials

The following supporting information can be downloaded at: https://www.mdpi.com/article/10.3390/ijms26083762/s1. References [4,19,20,21,24,32,33,34,35,36] are cited in Table S1.
