# Supplementary material for: Obstructive Sleep Apnea and Parkinson’s Disease: Bidirectional Clinical and Pathophysiologic Links
Source: Int J Mol Sci. 2025 Apr 16;26(8):3762. doi: 10.3390/ijms26083762 (PMC12028076; doi:10.3390/ijms26083762)
Supplement: Supplementary file 1 [file ijms-26-03762-s001.zip › ijms-3518982-supplementary.pdf]

Supplementary Table S1: Clinical association studies

Author, year	Study type	Population	Sample size	Study methodology	Significant results	Key findings	Conclusions
Zhu et al., 2024 [1]	Retrospective cohort study	PD patients with OSA	489	Patients were categorized using Unified Parkinson's Disease Rating Scale (UPDRS) and questionnaire into: Non-hallucinators (NVHs), minor visual hallucinations (MVHs) and formed visual hallucinations (FVHs). PSG was conducted to assess sleep parameters.	PD patients with visual hallucinations had a higher prevalence of OSA (63.3% in formed VHs vs. 38.7% in non-hallucinators), significantly higher AHI scores, and lower sleep efficiency ($p < 0.05$).	PD patients with OSA had a higher apnea-hypopnea index (AHI) and more severe oxygen desaturation than those without PD, exacerbating sleep fragmentation and daytime fatigue.	OSA severity is worsened by PD, with significant impacts on oxygenation and sleep quality.
Maggi et al., 2024 [2]	Meta-Analysis	PD patients with OSA	993	A systematic review of studies evaluating the prevalence of OSA in PD and its clinical correlates. OSA prevalence, apnea hypopnea index (AHI), and other factors were analysed	OSA was present in 66% of PD patients. PD patients exhibited significantly higher AHI scores compared to controls. Dopaminergic therapy showed no consistent influence on OSA severity.	OSA prevalence in PD is as high as up to 66%, driven by upper airway dysfunction and shared pathogenic mechanisms	There is significant prevalence of OSA in PD, necessitating early detection and treatment
Jeon et al., 2023 [3]	Retrospective Cohort Study	All OSA patients	29469	Data from the Korean National Health Information Database was used. Patients were matched with controls based on age, sex, and socioeconomic status. Diagnoses of OSA and PD were based on ICD-10 codes, and Cox proportional hazards regression models were used to calculate hazard ratios.	OSA patients had a 1.54-fold higher incidence of PD compared to non-OSA controls (HR 1.54, 95% CI 1.14-2.07, $p < 0.05$).<br><br>PD patients had a 1.92-fold higher incidence of OSA compared to non-PD controls (HR 1.92, 95% CI 1.32-2.78, $p < 0.05$).	OSA patients with concurrent PD exhibited worse apnea severity and higher mortality rates compared to those without PD.	A bidirectional relationship where PD worsens OSA outcomes
Aarsland et al., 2021 [4]	Review article	PD patients	NA	Review including clinical studies, biomarker research, and imaging data specifically examining epidemiology, pathophysiology, diagnosis, and treatment of cognitive impairment in PD.	Cognitive impairment affects up to 75% of PD patients within 10 years, with executive and visuospatial deficits as early markers and memory impairment in advanced stages. Biomarkers like cortical atrophy and Lewy body	OSA is associated with greater cognitive impairment from mild deficits to severe dementia in existing PD patients	Non-motor symptoms like cognitive impairment share underlying pathophysiologica l mechanisms with OSA

					pathology are key contributors to cognitive decline.		
Bargiotas et al., 2021 [5]	Cross-Sectional Study	PD patients undergoing DBS	50	PSG done to diagnose OSA. Motor, non-motor, and quality of life outcomes assessed pre- and post-subthalamic nucleus (STN) deep brain stimulation.	PD patients found that 44% had mild OSA, with significantly worse motor symptom scores (UPDRS-III OFF: 41.1 vs. 30.9, $p < 0.05$). Post-DBS outcomes were unaffected by OSA status or CPAP.	PD patients with OSA exhibited increased motor symptom severity compared to those without OSA	OSA in PD patients worsens motor outcomes
Elfil et al., 2021 [6]	Systematic review	Adult patients with PD and OSA	783	Systematic review evaluating the impact of OSA on cognitive and motor functions in PD.	OSA prevalence up to 60% in PD patients with moderate-to-severe OSA noted in 30%-40% of the PD population. Worsened cognitive function (MoCA ~15% lower) and motor severity (UPDRS-III ~20% higher) in PD patients.	OSA is associated with increased severity of PD-associated cognitive dysfunction and motor symptoms	OSA and PD have a bidirectional relationship each contributing to the pathophysiology of the other
Shen et al., 2020 [7]	Cross-Sectional Study	Chinese PD patients	239	Polysomnography (PSG) was used to diagnose OSA patients, dividing them into PD-OSA and control groups. Non-motor symptoms (NMS) and daytime sleepiness (Epworth Sleepiness Scale) was assessed while identifying risk factors for OSA.	OSA prevalence at 27.62% in PD patients, with age and male gender as risk factors, and RBD as protective. OSA worsened daytime sleepiness (higher ESS scores, $p < 0.05$), but was not linked to other non-motor symptoms.	OSA prevalence in PD patients was significant linked with excessive daytime sleepiness seen in PD	OSA exacerbates excessive daytime somnolence in PD
Meng et al., 2020 [8]	Longitudinal Study	PD patients	67	PD patients was stratified by OSA severity and CPAP use, with motor function assessed using mUPDRS and TUG over 12 months. Mixed models adjusted for demographics, BMI, levodopa dose, and comorbidities evaluated the impact of CPAP therapy on motor outcomes.	OSA was associated with worse motor scores (mUPDRS 24.5 vs. 16.2, $p < 0.001$).<br><br>CPAP therapy stabilized motor function (mUPDRS $\beta = -0.01$, $p = 0.03$) and improved mobility (TUG $\beta = -0.01$, $p = 0.002$) compared to the other groups.	CPAP use in OSA/PD patients were was associated with stable motor function and improved mobility	OSA in PD patients can worsen the progression of motor symptoms

Sobreira-Neto et al., 2019 [9]	Cross-Sectional Study	PD patients	88	Analysis of PD patients who underwent clinical interviews, standardized sleep scales (ESS, PSQI), and video-PSG to diagnose OSA and logistic regression identified associations between OSA and demographic or clinical factors.	The study found that 66% of PD patients had OSA and had significantly worse sleep parameters (lower N3 sleep, higher arousal index, $p < 0.05$). Supine position was an important factor associated with 58% of OSA cases in PD patients.	OSA was associated with reduced slow-wave sleep, higher oxygen desaturation index, and worsened arousal index in PD patients	Supports the importance of early detection and treatment of OSA in PD to prevent worsening of sleep-related symptoms.
Mery et al., 2017 [10]	Retrospective Cohort Study	PD patients	67	PD patients was diagnosed with OSA using PSG) to diagnose OSA. Standardized scales like MoCA and ESS were used to assess cognitive function and daytime sleepiness. Regression models evaluated associations between OSA severity, cognitive decline, and sleep-related arousals.	61.6% of PD patients had concurrent OSA. Cognitive function (MoCA) was 5.2% lower ($p = 0.043$) and daytime sleepiness (ESS) 6.7% higher ($p = 0.031$) in OSA patients. Sleep-related arousals were also negatively associated with cognitive performance ($p = 0.049$).	OSA was associated with cognitive dysfunction, including worse MoCA scores in PD patients	OSA could be a modifiable risk factor for cognitive decline in PD

1. Zhu, J.; Zhao, Y.; Jiang, Y.; Pan, Y.; Jiang, X.; Wang, Y.; Li, D.; Zhang, L. The relationship between obstructive sleep apnea and visual hallucinations in PD patients: a polysomnography study. *Front. Neurol.* **2024**, *14*, 1275660, https://doi.org/10.3389/fneur.2023.1275660.
2. Maggi, G.; Giacobbe, C.; Iannotta, F.; Santangelo, G.; Vitale, C. Prevalence and clinical aspects of obstructive sleep apnea in Parkinson disease: A meta-analysis. *Eur. J. Neurol.* **2024**, *31*, e16109. https://doi.org/10.1111/ene.16109.
3. Jeon, S.-H.; Hwang, Y.S.; Oh, S.-Y.; Shin, B.-S.; Kang, M.G.; Lee, M.G.; Yeom, S.W.; Lee, J.H.; Kang, H.G.; Kim, J.S. Bidirectional association between Parkinson's disease and obstructive sleep apnea: a cohort study. *J. Clin. Sleep Med.* **2023**, *19*, 1615–1623, https://doi.org/10.5664/jcsm.10596.
4. Aarsland, D.; Batzu, L.; Halliday, G.M.; Geurtsen, G.J.; Ballard, C.; Chaudhuri, K.R.; Weintraub, D. Parkinson disease-associated cognitive impairment. *Nat. Rev. Dis. Primer.* **2021**, *7*, 1–21, https://doi.org/10.1038/s41572-021-00280-3.
5. Bargiotas, P.; Bargiotas, I.; Debove, I.; Lachenmayer, M.L.; Vayatis, N.; Schuepbach, W.M.; Bassetti, C.L. Sleep apnea syndrome and subthalamic stimulation in Parkinson's disease. *Sleep Med.* **2021**, *86*, 106–112, https://doi.org/10.1016/j.sleep.2021.07.031.
6. Elfil, M.; Bahbah, E.I.; Attia, M.M.; Eldokmak, M.; Koo, B.B. Impact of Obstructive Sleep Apnea on Cognitive and Motor Functions in Parkinson's Disease. *Mov. Disord.* **2021**, *36*, 570–580, https://doi.org/10.1002/mds.28412.
7. Shen, Y.; Dong, Z.-F.; Pan, P.-L.; Shi, H.-C.; Liu, C.-F. Obstructive sleep apnea in Parkinson's disease: a study in 239 Chinese patients. *Sleep Med.* **2020**, *67*, 237–243, https://doi.org/10.1016/j.sleep.2019.11.1251.
8. Meng, L.; Benedetti, A.; Lafontaine, A.-L.; Mery, V.; Robinson, A.R.; Kimoff, J.; Gros, P.; Kaminska, M. Obstructive sleep apnea, CPAP therapy and Parkinson's disease motor function: A longitudinal study. *Park. Relat. Disord.* **2020**, *70*, 45–50, https://doi.org/10.1016/j.parkreldis.2019.12.001.
9. Sobreira-Neto, M.A.; Pena-Pereira, M.A.; Sobreira, E.S.T.; Chagas, M.H.N.; de Almeida, C.M.O.; Fernandes, R.M.F.; Tumas, V.; Eckeli, A.L. Factors related to excessive sleepiness in patients with Parkinson's disease. *Neurol. Res.* **2019**, *41*, 227–233, https://doi.org/10.1080/01616412.2018.1548746.
10. Mery, V.P.; Gros, P.; Lafontaine, A.-L.; Robinson, A.; Benedetti, A.; Kimoff, R.J.; Kaminska, M. Reduced cognitive function in patients with Parkinson disease and obstructive sleep apnea. *Neurology* **2017**, *88*, 1120–1128, https://doi.org/10.1212/wnl.0000000000003738.
